# Memorable Audiovisual Narratives Synchronize Sensory and Supramodal Neural Responses

**DOI:** 10.1523/ENEURO.0203-16.2016

**Published:** 2016-11-10

**Authors:** Samantha S. Cohen, Lucas C. Parra

**Affiliations:** 1 Department of Psychology, The Graduate Center, City University of New York, New York, New York 10016; 2 Department of Biomedical Engineering, City College of New York, New York, New York 10031

**Keywords:** episodic encoding, intersubject correlation, multisensory integration, naturalistic stimuli

## Abstract

Our brains integrate information across sensory modalities to generate perceptual experiences and form memories. However, it is difficult to determine the conditions under which multisensory stimulation will benefit or hinder the retrieval of everyday experiences. We hypothesized that the determining factor is the reliability of information processing during stimulus presentation, which can be measured through intersubject correlation of stimulus-evoked activity. We therefore presented biographical auditory narratives and visual animations to 72 human subjects visually, auditorily, or combined, while neural activity was recorded using electroencephalography. Memory for the narrated information, contained in the auditory stream, was tested 3 weeks later. While the visual stimulus alone led to no meaningful retrieval, this related stimulus improved memory when it was combined with the story, even when it was temporally incongruent with the audio. Further, individuals with better subsequent memory elicited neural responses during encoding that were more correlated with their peers. Surprisingly, portions of this predictive synchronized activity were present regardless of the sensory modality of the stimulus. These data suggest that the strength of sensory and supramodal activity is predictive of memory performance after 3 weeks, and that neural synchrony may explain the mnemonic benefit of the functionally uninformative visual context observed for these real-world stimuli.

## Significance Statement

Although multisensory integration is an important part of daily life, the mnemonic influence of one modality on another is not well established. Cross-modal cues may either strengthen or interfere with memory for information imparted through another sensory modality. We establish that during the encoding of a naturalistic auditory stimulus the cross-subject synchrony of neural processing predicts memory performance regardless of stimulus modality. The dominant neural signature of enhanced encoding is supramodal in that it is largely independent of the modality of stimulus presentation. The level of synchrony that a story elicits may help to predict the extent to which adding extraneous information benefits memory.

## Introduction

It is often easier to remember your friends’ stories when they illustrate them with photos. These multisensory representations of the world can facilitate encoding by providing multiple cues regarding the salience of experienced events (Giard and Peronnet, 1999). By some accounts, the primary role of the brain is as a multisensory integrator. However, this does not necessarily mean that additional sensory information will enhance encoding. The content relayed through simultaneous auditory and visual streams can either strengthen or interfere with unisensory memory (Shams and Seitz, 2008). A supplementary modality usually enhances memory when it is semantically congruent with the primary stimulus; however, it can be detrimental if it does not impart meaningfully relevant information (von Kriegstein and Giraud, 2006; Cohen et al., 2009; Matusz et al., 2015). Benefits are often ascribed to an associative memory mechanism, whereby memories from one modality can cue the retrieval of those imparted by another (Fuster, 1997). Decrements are explained using theories of limited attention that posit that superfluous modalities may distract from the learning of pertinent information (Murdock, 1965; Craik et al., 1996). There is little certainty as to which mechanism will dominate in any given situation. Additionally, most existing research on multisensory memory addresses memory for discrete stimuli, rather than the semantic aspects of dynamic everyday experiences.

Arguably, the benefits or detriments of the added modality will depend on its effects on the neural processing of the stimulus during encoding. It is well established that memory accuracy can be predicted by evoked response magnitude during encoding (Brewer et al., 1998; Wagner et al., 1998; Kim, 2011). Yet, very little is known about the neural substrate of multisensory memory effects in a naturalistic context. The magnitude of responses evoked by discrete multisensory stimuli has previously been linked to memory (Murray et al., 2004; Thelen et al., 2012, 2014; Altieri et al., 2015; Matusz et al., 2015). However, there is no similar evidence regarding how the neural processing of multisensory stimuli potentiate memory in the context of naturalistic, contextually rich stimuli that can be understood from a single modality.

We hypothesized that the synchrony of neural responses between individuals attending to the same naturalistic multisensory stimulation is predictive of memory. Discrete regions of cortex have been shown to exhibit enhanced intersubject correlation (ISC) in fMRI during the encoding of successfully remembered items from a narrative (Hasson et al., 2008). However, it has not yet been determined whether ISC, measured on the fast timescale of electrophysiology (<1 s), can be used as a surrogate for the successful encoding of a dynamic narrative stimulus. Therefore, to quantify the reliability of evoked responses, we measure the ISC of neural activity across the group experiencing the stimulus, following work in fMRI, electroencephalography (EEG), and MEG (Hasson et al., 2004; Dmochowski et al., 2012; Lankinen et al., 2014). In contrast to previous work, the focus here is on multisensory memory effects, and thus synchrony of neural activity is assessed for auditory, visual, and audiovisual stimuli.

We expected that visuals enhance engagement with the stimulus and, thus, potentiate the encoding of auditory information. EEG was recorded during the presentation of biographical narratives, and the memory for auditorily imparted story elements was tested 3 weeks later in an effort to mimic the features of real-world episodic encoding. The narratives were presented solely auditorily, or combined with illustrative visual animations. ISC was used to assess encoding efficacy because it is indicative of attention and preference (Dmochowski et al., 2014; Ki et al., 2016), and is therefore likely representative of enhanced stimulus processing. Although the visual stimulus alone did not induce any meaningful memory, it improved retrieval when combined with the narrative, regardless of whether it was temporally aligned with the audio. Additionally, the synchrony of stimulus-evoked neural processing across individuals was predictive of memory. The spatial distribution of this predictive neural activity was largely consistent across auditory and visual stimuli. Thus, under realistic conditions, functionally uninformative visual content enhances both subsequent memory and intersubject correlation of supramodal evoked response.

## Materials and Methods

### Participants

A total of 88 fluent English-speaking subjects (age, 25 ± 6 years; 23 females) with normal or corrected-to-normal vision participated in the experiment. Of the original 88 subjects, 75 completed the follow-up memory assessment 3 weeks after stimulus presentation. All participants provided written informed consent, and were remunerated for their participation. Additionally, they all had self-reported little to no familiarity with the stimulus. Procedures were approved by the Institutional Review Board of the City University of New York.

### Stimuli presentation

**Figure 1.**
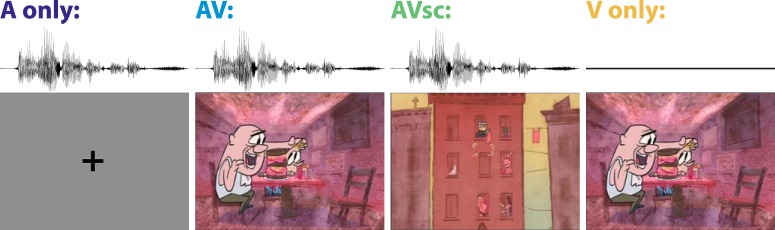
Illustration of the behavioral task. Subjects were exposed to one of five conditions: A Only, AV, AVsc, V Only, or No Stim (not shown). The sound clip represented by the waveform is “…and he would buy me a hotdog the size of my head…” Three weeks after stimulus presentation, and without prior warning, subjects were asked to complete an on-line questionnaire with 72 four-alternative forced-choice questions. The question asked about this segment of the stimulus was “What would Rocco do with the narrator when they went for walks?” Answer options were as follows: “a. Buy him a hot dog; b. Buy him a milkshake; c. Buy him candy; d. Tell him stories.” Still images from “Sundays at Rocco’s,” a StoryCorps animated short produced by Lizzie Jacobs and Mike Rauch, reproduced here with permission from StoryCorps.

The stimuli used were taken from 10 different videos [5 from the New York Times “Modern Love” episodes: “Broken Heart Doctor” (BHD), “Don’t Let it Snow” (DLIS), “Falling in Love at 71” (FILSO), “Lost and Found” (LF), and “The Matchmaker” (TM); and 5 from StoryCorps animated shorts: “Eyes on the Stars” (EOTS), “John and Joe” (JJ), “Marking the Distance” (MTD), “Sundays at Rocco’s” (SAR, depicted in Fig. 1), and “To R.P. Salazar with Love” (TRPSWL)]. The clips were on average 161 ± 44 s in length, and individual scenes were on average 17.9 ± 12.8 s in length. Scene duration differed significantly across videos (*F*_(9,76)_ = 1.98, *p* = 0.05). The audiovisual (AV), audio with scrambled visuals (AVsc), and audio only (A Only) versions of all stimuli are available at http://www.parralab.org/isc/memory-videos.html. The stories were chosen on the basis of their highly emotive content, which is thought to drive synchronous responses across subjects (Dmochowski et al., 2012). In addition to some music, the auditory component of each video consisted of a narration that could be understood without the accompanying animations. Subjects were in one of the following stimulus conditions. In the A Only condition, subjects listened to the sound from the video while their eyes fixated on a cross centered on a screen with a constant luminance equal to the mean across the 10 videos (*n* = 16, 13 completed memory battery). Prior to the onset of the auditory narration, introductory text, present in all conditions, was displayed to assure that all subjects had a consistent narrative context. In the AV condition, subjects watched the unadulterated videos (*n* = 21, 16 completed memory battery). In the AVsc condition, subjects watched videos where the auditory component was unchanged, but the scenes of the animations were randomly scrambled at scene cuts occurred 6–12 times per clip (*n* = 17, 14 completed memory battery). In the visual only (V Only) condition, subjects watched the silent animations without the auditory content (*n* = 18, 16 completed memory battery). In the no stimulus exposure (No Stim) condition, subjects were never presented with any stimuli nor was EEG collected, and they therefore answered the memory questionnaire naively (*n* = 16; 16 subjects completed the memory battery). Stimuli were edited according to stimulus condition with Lightworks software (EditShare EMEA 2014) and were presented in a random order, counterbalanced across conditions, using an in-house modified version of M-Player software (http://www.mplayerhq.hu), which provided trigger signals for the EEG acquisition system once per second during the duration of each stimulus, with a temporal jitter of less than ±2 ms across subjects. Stimuli were presented in a dark, and electrically and acoustically shielded room, with brief breaks between clips (<30 s).

### Memory test

Subjects were informed after stimulus presentation that they might be contacted for future correspondence regarding the stimuli. Three weeks later, without prior knowledge of a memory requirement, subjects received a memory test with four-alternative forced-choice questions (*n* = 72) presented via LimeSurvey (LimeSurvey Project Team/Carsten Schmitz, 2012), where five to nine questions corresponded to each story. The order of the questions concerning each story matched the order in which the stories had been originally presented to each participant. The questions concerned information that could be acquired entirely through the A Only presentation. The content of the questions was either of a factual nature, which was literally stated during the narrative (three to eight questions per story; e.g., “What would Rocco do with the narrator when they went for walks?”; Fig. 1), or concerned emotional content that could only be learned through theory of mind reasoning (one to two questions per story; e.g., “How did the narrator feel about the apartment building being condemned?” (Frith and Frith, 1999).

### EEG data collection and preprocessing

The EEG was recorded with a BioSemi Active Two system at a sampling frequency of 512 Hz. Subjects were fitted with a standard, 64-electrode cap following the international 10/10 system. To subsequently remove eye-movement artifacts, the electrooculogram (EOG) was also recorded with six auxiliary electrodes (one each located dorsally, ventrally, and laterally to each eye). All signal processing was performed off-line in the MATLAB software (MathWorks).

Data preprocessing procedures followed those in the study by Dmochowski et al. (2012). The EEG and EOG data were first downsampled to 256 Hz, high-pass filtered (1 Hz cutoff), and notch filtered at 60 Hz. After extracting the EEG/EOG segments corresponding to the duration of each stimulus, electrode channels with high variance were manually identified and replaced with zero-valued samples using visual inspection, effectively discounting these channels in the subsequent calculation of covariance matrices. Eye-movement artifacts were removed by linearly regressing the EOG channels from the EEG channels. Outlier samples were identified in each channel (magnitude exceeded 3 SDs of the mean of their respective channel), and samples 40 ms before and after such outliers were replaced with zero-valued samples. These stringent artifact rejection techniques were used due to the sensitivity to outliers of the covariance matrices used in the neural synchrony computation.

### Intersubject correlation

To determine the fidelity with which a unique stimulus presentation is processed, the ISC of the neural responses is computed. The correlation of responses between subjects is similar to that of traditional evoked response analyses in that both measures increase in magnitude when responses are reliably reproduced (either across subjects or trials). They are also similar measures in that, in order to find correlation between subjects, responses must be reliable within each individual (Hasson et al., 2009). In the present circumstances, where repeatedly presenting an identical stimulus to the same subject would artificially potentiate their memory, the ISC metric has a particular advantage over traditional evoked response analyses. Fortunately, in a naturalistic setting, where stimuli occur in a continuous stream, ISC can be assessed by fMRI, EEG, and MEG (Hasson et al., 2004; Dmochowski et al., 2012; Lankinen et al., 2014). Here we use EEG in order to measure the correlation between fast stimulus-evoked responses across subjects. “Fast” means that these stimulus-evoked responses, high-pass filtered at 1 Hz, are faster than the hemodynamic response for fMRI. ISC is evaluated in the correlated components of the EEG and can be measured with as few as 12 subjects (Dmochowski et al., 2012, 2014). The goal of correlated component analysis in this case is to find linear combinations of electrodes (one could think of them as virtual sensors or “sources” in the brain) that are consistent across subjects and maximally correlated between them.

Correlated component analysis is similar to traditional principal component analysis except that it extracts projections of the data with maximal correlation rather than maximal variance. The technique requires calculation of the pooled between-subject cross-covariance, $R_{b}=\frac{1}{N(N-1)}\sum_{k}\sum_{l,l\neq k}R_{kl}$, and the pooled within-subject covariance, $R_{w}=\frac{1}{N}\sum_{k}R_{kk}$, where $R_{kl}=\underset{t}{\sum }(x_{k}(t)-{\overset{¯}{x}}_{k}){\left(x_{l}(t)-{\overset{¯}{x}}_{l}\right)}^{\text{T}}$ measures the cross-covariance of all electrodes in subject *k* with all electrodes in subject *l*. Vector $x_{k}(t)$ represents the scalp voltages at time *t* in subject *k*, and, ${\bar{x}}_{k}$, their mean value in time. The component projections that capture the largest correlation between subjects (i.e., the ISC) are the eigenvectors *v_i_* of matrix $R_{w}^{-1}R_{b}$ with the strongest eigenvalues, which measure the strength of correlation in the *i*th component, as follows:

(1) $$C_{i}=\frac{v_{i}^{T}R_{b}v_{i}}{v_{i}^{T}R_{w}v_{i}}\;.$$

High ISC is obtained when the responses are similar across subjects. Prior to computing eigenvectors, the pooled within-subject correlation matrix is regularized in order to improve robustness to outliers using shrinkage (Blankertz et al., 2011). Between-subject and within-subject covariance matrices were computed for all subjects in each stimulus condition, regardless of whether the memory questionnaire was completed. These matrices were subsequently averaged over the 10 stimuli and over all presentation modalities (A Only, V Only, AV, and AVsc) to obtain a common set of components applicable to all conditions. Note that the covariance matrices are normalized by the number of subjects in each condition so that the unequal number of subjects in each condition does not bias the results. Additionally, the matrices for AV and AVsc were first averaged together prior to combining with the other modalities so as not to bias results by the two repeated multisensory conditions.

The same component projections *v_i_* were therefore used for all stimulus conditions to measure ISC. The three strongest correlated components were selected, and the corresponding correlation values were computed separately for each condition, each component, and each of the 10 narratives. The ISC is reported as the correlation summed over all components, as follows: $ISC=\sum_{i}C_{i}$. This is limited to the strongest three components so that the neural metrics reported measure the overall level of synchrony evoked by the stimulus regardless of anatomical origin. Additionally, correlations *C_i_* in the weaker components were not always significantly different from chance (phase shuffle statistics; see below and Fig. 3*A*, where gray indicates the phase-shuffled ISC), and the spatial distributions of these weaker components differed across modality.

To determine how similar each subject is to the others experiencing the same stimulus, the ISC is computed on an individual subject basis. The correlation is computed between a given subject, *k*, and all others who experienced the same condition, as follows:

(2) $$C_{ik}=\frac{v_{i}^{T}R_{b,k}v_{i}}{v_{i}^{T}R_{w,k}v_{i}}\;,$$

using the following definitions for the between-subject and within-subject covariance: $R_{b,k}=\frac{1}{\left(N-1\right)}\sum_{l,l\neq k}\left(R_{kl}+R_{lk}\right)$, and $R_{w,k}=\frac{1}{\left(N-1\right)}\sum_{l,l\neq k}\left(R_{kk}+R_{ll}\right)$, which are symmetrized to ensure a proper normalization as a correlation coefficient. The ISC per subject is defined again as the sum of correlation across components, as follows:$ISC_{k}=\sum_{l,l\neq k}C_{ik}$. To resolve a common set of components for the different conditions, the projection vectors are computed using the average of the correlation matrices across conditions. Note, however, that the ISC for individual subjects using these projection vectors is then computed only within condition (i.e., by measuring the reliability of responses only between subjects exposed to the identical stimulus). To rule out the possible dependence of the ISC measure between conditions, we repeated this analysis using projections *v_i_*, which maximize the correlations within conditions.

ISC values that can be obtained by chance were determined by computing the ISC in a manner identical to that described above (including component extraction), using 100 renditions of surrogate data following the procedure of Prichard and Theiler (1994). By randomizing phase identically in all channels, these surrogate data perturb the time course of the data but preserve the temporal and spatial correlation in the original EEG signal. Significance tests were corrected for multiple comparisons while controlling the false discovery rate (Benjamini and Hochberg, 1995).

To visualize the spatial distribution of the component activity, the “forward model” is computed for each component (Parra et al., 2005; Haufe et al., 2014). A forward model represents the covariance between each component’s activity and the activity at each electrode location. To provide a meaningful scale, we deviate from the literature by normalizing this covariance by the signal magnitudes to indicate correlation coefficients that scale between −1 and +1. The code to compute the ISC is available at http://www.parralab.org/isc/.

### Comparisons of both memory accuracy and intersubject correlation

Due to the between-subjects design, all statistical comparisons (ANOVAs, *t* tests, and *z*-tests) are unpaired, unless otherwise stated (e.g., in cases where comparisons are made between the same memory questions asked to different groups of subjects). ANOVAs to assess differences in memory accuracy across conditions were computed using the accuracy value for each question averaged across subjects. To assess the nontrivial correlation between ISC and memory accuracy, the effect of stimulus modality was controlled for as both variables were strongly and significantly modulated by the addition of visuals to the auditory component. When assessing the correlation across subjects, the mean for each condition was subtracted from each individual’s ISC and memory accuracy. In addition to accounting for stimulus modality, correlations included only values from conditions where memory performance was above chance (assessed via comparison with the No Stim condition); the V Only ISC was therefore not related to memory that was not tested for.

### Strength of oscillatory activity

For the oscillatory power analysis, the frequency bands that have previously been associated with memory and attention (theta, alpha, and gamma) were used. For each subject, band power was calculated in both individual electrodes and in each of the correlated components. Band-pass powers were then then individually normalized by the total broadband power and then averaged across narratives. Power was measured on the band-passed signals for theta (4.5–9.5 Hz), alpha (7.5–12.5 Hz), and gamma (30–50 Hz) frequency bands using a Morlet filter.

## Results

We sought to investigate whether the ISC of electroencephalographic evoked responses is predictive of memory for auditory information in the context of realistic multisensory episodic memory. Ten biographical narratives were presented to separate groups of individuals who either solely heard the stories (A Only), or heard them with accompanying visual animations that complemented the auditory narrative (AV). This between-subjects design allowed a comparison between unisensory and multisensory stimulation while avoiding confounds, such as memory potentiation, due to repeated stimulus presentations. To determine the importance of semantic congruency, a control group heard the story with the same visual animations scrambled in time so that they did not semantically match the auditory stream (AVsc). To measure the information content of the visual stimulus alone, another control group watched the visual animations without the narration (V Only; Fig. 1, illustration). The chance-level performance on the question battery was measured in a third control group, who answered the memory questions without experiencing either the auditory or the visual stimulus (No Stim). To assess incidental episodic memory, subjects were not aware that they would be asked to retrieve the information presented in the auditory narration 3 weeks later. EEG activity was measured in 72 subjects during stimulus presentation to assess neural processing during encoding. We expected that the supplementary visuals would boost memory performance when congruent with the auditory stories (AV, but not AVsc). We also hypothesized that when the auditory narrative was present (A Only, AV, and AVsc, but not V Only), the accuracy with which subjects remembered the stories would be predicted by how correlated their brain activity was to others responding to the same stimulus.

### Multisensory presentations enhance incidental episodic memory

A 72-question evaluation assessed memory for auditory content from the 10 narratives. Subjects who heard the stories (A Only, AV, or AVsc) correctly answered 70.1 ± 21.8% of the questions, a level significantly above chance performance (*t*_(71)_ = 15.0, *p* = 1e-23; all *t* tests in this section are paired samples *t* test across questions) established in subjects who were naive to the stimulus (No Stim condition, 35.6 ± 18.8%). Nine questions in the No Stim condition were answered at a level above numerical chance (25%, determined via one sample *z*-tests for proportions and FDR corrected for multiple comparisons).

In contrast to the conditions containing the auditory narrative, performance in the V Only condition (37.2 ± 24.1%) was indistinguishable from chance (*t*_(71)_ = 0.7, *p* = 0.5, paired samples *t* test across questions) and was, thus, functionally uninformative. Therefore, as intended, the visual stimulus did not carry any meaningful, question-pertinent information (with the exception of one question answered significantly better by V Only participants than by No Stim participants, determined via two-sample *z*-tests for proportions and FDR corrected). It is possible that had the questions also probed for visual information, the differences between audio and visual memory performance would have been different. Subsequent memory analyses will therefore examine memory performance only for conditions in which the auditory narrative was presented (A Only, AV, and AVsc).

**Figure 2.**
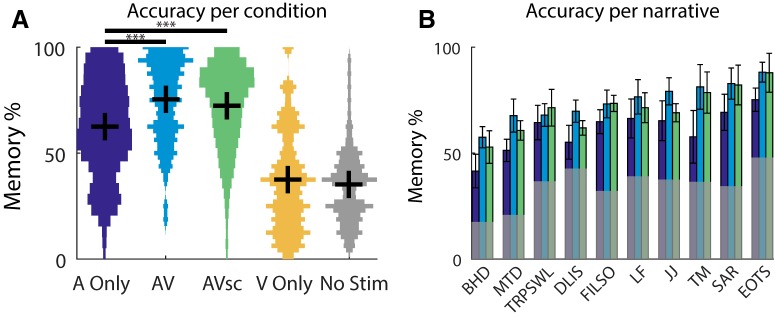
***A***, ***B***, Memory performance for different stimulus modalities (***A***) and different narratives (***B***). ***A***, Note that exposure to the visual stimuli (V Only, yellow) yields performance no better than chance performance (No Stim, gray). In addition to mean and SE (represented by the black horizontal and vertical lines, respectively), we also present the histogram of the distribution of accuracy values. ***B***, For each narrative (for titles see Materials and Methods), performance is shown for A Only (purple), AV (blue), AVsc (green), and chance (No Stim, gray). Error bars represent the SEM across questions (*N* = 72 in ***A***; *N* = 5–9 in ***B***). *p < 0.05, **p < 0.01, ***p < 0.001.

A two-way repeated-measures ANOVA for memory accuracy, with condition as a factor (A Only, AV, and AVsc) and narrative as a repeated-measures factor, revealed a significant effect of condition (*F*_(2,186)_ = 35.0, *p* = 6e-8; Fig. 2*A*) and narrative (*F*_(9,186)_ = 20.6, *p* < 1e-7; Fig. 2*B*), but no interaction (*F*_(18,186)_ = 0.2, *p* = 1, ANOVA calculated on the accuracy value of each question, averaged across subjects). A repeated-measures ANOVA was used here because the memory questions used for each narrative were the same, or repeated, across conditions. Additionally, there was no significant effect of who produced the narrative (*F*_(1,186)_ = 20.2, *p* = 0.07, determined via a nested two-way ANOVA contrasting New York Times-produced and StoryCorps-produced stories), and production did not interact with condition (*F*_(2,186)_ = 0.1, *p* = 0.9). Although the visual stimulus alone did not carry any question-related information, the effect of condition was driven by a significant boost in memory performance when the visual stimulus was combined with the auditory narrative. This effect holds even when the visual stimulus was incongruent with the story [12.9 ± 16.1% improvement above A Only for AV (*t*_(71)_ = 6.8, *p* = 3e-9); and 9.6 ± 16.6% improvement above A Only for AVsc (*t*_(71)_ = 4.9, *p* = 6e-6); Fig. 2*A*]. The congruent visual stimulus enhanced memory slightly better than the incongruent stimulus (AV vs AVsc, *t*_(71)_ = 2.1, *p* = 0.04).

The variation in performance across narratives may indicate that question difficulty varied across stories as a result of experimenter bias. However, information retrieval varied across narratives even after controlling for the variation in chance-level performance (*F*_(9,186)_ = 9.6, *p* = 3e-5; performance on each question in the No Stim condition was subtracted prior to the ANOVA; Fig. 2*B*). This may indicate that some stories were genuinely more memorable than others. Furthermore, the lack of an interaction between narrative and condition suggests that the visual boost in memory performance generalizes across stories and was not specific to the content of the animations.

### Multisensory presentations increase the synchrony of neural responses

**Figure 3.**
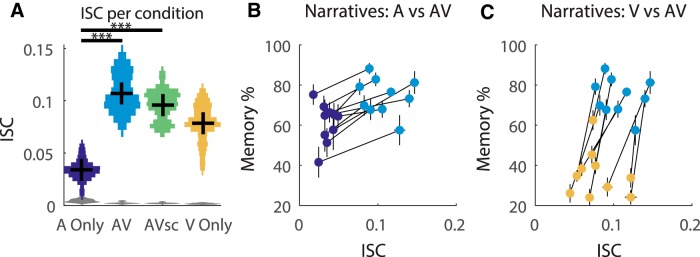
ISC A Only (purple), AV (blue), AVsc (green), and visual (V Only, yellow) stimuli. The full distribution of the ISC values are indicated by the width of the histogram bars for each condition, and gray indicates the distribution of the chance level of correlation for each modality. ISC is calculated using the sum of the three largest correlated components elicited by the presentation of the narrative (Eq. 1). Error bars (vertical lines) represent the SEM across subjects. *p < 0.05, **p < 0.01, ***p < 0.001. ***B***, ***C***, The multisensory boost in memory and ISC occurs for all 10 narratives. The different presentation conditions for each narrative, corresponding to separate groups of subjects, are connected with a line, and SEs across subjects are represented as horizontal and vertical bars for ISC and Memory %, respectively.

The ISC is measured following previous research (Dmochowski et al., 2012; Ki et al., 2016; Eq. 1). An ANOVA comparing the ISC across conditions revealed a significant effect of condition (*F*_(3,68)_ = 66.4, *p* = 9e-20). The visual animations (V Only) evoked stronger ISC than those evoked by the auditory narrative alone (Fig. 3*A*; A Only vs V Only, *t*_(31)_ = 8.4, *p* = 2e-9). This is not unexpected, given that a large fraction of cortex is dedicated to visual processing (Felleman and Van Essen, 1991). Adding a second modality to the unimodal stimuli increases the ISC (AV vs V Only, *t*_(36)_ = 4.8, *p* = 2e-5; and AV vs A Only, *t*_(33)_ = 14.0, *p* = 2e-15), and adding visual stimulation to the auditory story increases ISC, even when the visual stimulus is temporally incongruent (AVsc vs V Only, *t*_(32)_ = 3.1, *p* = 0.004; and AVsc vs A Only, *t*_(29)_ = 13.7, *p* = 4e-14). Additionally, AV has a slightly higher ISC than does AVsc (*t*_(34)_ = 2.1, *p* = 0.05). This is the same pattern of modulation observed for memory accuracy (Fig. 3*B*,*C*), and, with the exception of the weaker difference between AV and AVsc, these effects are preserved when the ISC is computed on each condition separately (*t*_(34)_ = 1.7, *p* = 0.09). Note that the effect of adding a modality is not expected to be additive in a numerical sense, either for memory performance, which has a strict ceiling, or for ISC, which is a measure of correlation and is, therefore, nonlinear.

If the multisensory enhancement in memory could be explained by the corresponding increase in ISC, we would expect that the boost in ISC (A vs AV) would correlate with the corresponding boost in memory (Fig. 3*B*). However, the relationship could not be resolved in this small sample (*r* = 0.20, *p* = 0.6, *N* = 10).

### Neural synchrony and memory for auditory information are correlated

**Figure 4.**
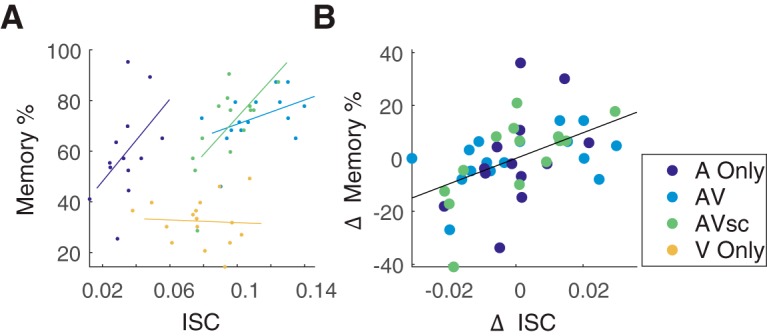
Relationship between neural ISC and memory performance. ***A***, Memory accuracy for auditory information increases with ISC in all conditions in which the auditory narrations were heard (A Only, AV, and AVsc), but not when it was missing (V Only). Each point indicates an individual subject’s ISC (Eq. 2) and memory. ***B***, Same as ***A***, but here, to control for the modality effect, mean values across subjects were subtracted from ISC and memory performance for each subject in that stimulus condition. Only conditions with performance significantly above chance are used.

We hypothesized that ISC predicts memory performance regardless of stimulus modality. Figure 4*A* shows the relationship between each individual’s ISC (Eq. 2) and their memory in all four stimulus conditions. This relationship is significant only in the AVsc condition (*r* = 0.67, *p* = 0.009, *N* = 14). The numerical value of the correlation is also positive in the other conditions in which the narrative was present, although the sample sizes may have been too small to resolve a significant effect (A Only: *r* = 0.48, *p* = 0.1, *N* = 13; AV: *r* = 0.43, *p* = 0.1, *N* = 16). This relationship is numerically negative for subjects who did not hear the narrative (V Only: *r* = −0.08, *p* = 0.8, *N* = 16). This was expected since the memory questionnaire assessed only auditorily imparted information (V Only memory performance was at chance level; Fig. 2*A*).

The addition of these visuals to the auditory story increased both ISC and memory. Therefore, to control for this multisensory boost in the conditions where auditory information was presented (A Only, AV, and AVsc), the mean values for each condition are subtracted from subjects in that condition yielding ΔISC and ΔMemory percentage values (Fig. 4*B*). Subjects whose neural responses were more synchronous with others remembered the stories more accurately (*r* = 0.49, *p* = 9e-4, *N* = 43 subjects). This relationship is similarly strong regardless of whether emotional or factual information was tested (*r* = 0.44, *p* = 0.002, for factual questions; *r* = 0.52, *p* = 3e-4, for emotional questions). Additionally, if the ISC components are chosen to maximize correlation within each condition, rather than in the average over conditions, ISC still predicts memory across subjects (*r* = 0.45, *p* = 0.002, *N* = 43 subjects).

One possible interpretation of this result is that attention modulates both ISC (Ki et al., 2016) and memory performance (Murdock, 1965; Craik et al., 1996), and this therefore induces the correlation between the two. To assess this, we consider an additional neural measure known to be modulated by attention: alpha power.

### Alpha activity modulated by modality but not correlated to memory performance

Attention is known to affect alpha-band power (Ray and Cole, 1985; Cooper et al., 2003; O’Connell et al., 2009), and alpha power decreases during encoding are correlated with memory performance (Klimesch et al., 1996; Hanslmayr et al., 2009). Similarly to the correlations computed for ISC, the correspondence between alpha power and memory was assessed for subjects and questions. Following previous research (Adrian and Matthews, 1934; Gale et al., 1971), and in agreement with the ISC, alpha power was significantly modulated by the addition of visual stimuli to the auditory narration (62 of 64 electrodes significantly decreased in power between A Only and AV conditions, on average, −2.6 ± 0.5 dB, *N* = 39 subjects; and 61 electrodes significantly decreased in power between A Only and AVsc conditions, on average, −2.0 ± 0.5 dB, *N* = 35 subjects; all comparisons were FDR corrected at *p* < 0.05 and computed by shuffling condition labels). However, unlike ISC, after accounting for the modality effect, no correspondence between alpha power and memory was found in any electrode or in the combination of electrodes most correlated across subjects (all comparisons were FDR corrected at *p* < 0.05). Although the relationship between ISC and memory may be driven by attention (Ki et al., 2016), performing a mediation analysis to establish a causal link between alpha and ISC, which accounts for the relationship of ISC to memory, was unsuccessful due to the fact that alpha power did not correlate with memory.

Since theta and gamma power have also been implicated in memory performance and maintenance over shorter timescales (Osipova et al., 2006; Sauseng et al., 2009; Fuentemilla et al., 2010), these analyses were repeated for the theta and gamma bands. No change in power was found when adding the visual stimulus to the auditory story. Additionally, neither band significantly correlated with memory performance (with the exception of a single electrode whose theta-band power correlated with memory accuracy in the across-questions analysis).

### Spatial distributions of synchronous neural response are preserved across modalities

**Figure 5.**
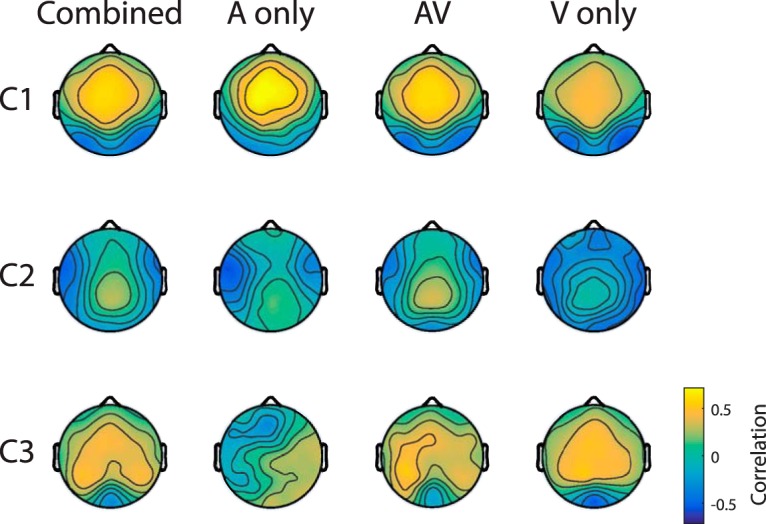
The forward model for the three most correlated components of neural activity. Each column represents the forward model (correlation between surface electrodes and component activity) obtained using either all stimuli together (combining responses across all subjects, left) or different stimulus presentations (A Only, middle-left; AV, middle-right; V Only, right). Each row represents a different component in descending order from most correlated (top) to least correlated (bottom; C1–C3). Color indicates the correlation between each scalp electrode and the component.

ISC is measured in components of the EEG that are maximally correlated between subjects (see Materials and Methods). Note that, by design, components are temporally uncorrelated with each other and thus capture different sources of neural activity. To visualize the spatial distribution of these different sources, a “forward model” is computed for each component (Parra et al., 2005; Haufe et al., 2014). The magnitude of the forward model represents the strength to which each scalp electrode contributes to that component. The sign indicates the sign of the evoked potentials at that location. First, in parallel with the ISC computations described above, data are combined from all conditions (A Only, AV, AVsc, and V Only; Fig. 5, combined column). Additionally, forward models are computed separately per condition to determine the stability of the correlated components in separate conditions (Fig. 5, AV, A Only, and V Only). The components for AVsc are not presented as they look identical to those for AV. The resulting distributions for the three largest correlated components in the AV condition are similar to previous results using AV stimuli (Dmochowski et al., 2012). The first two components have a similar spatial distribution across conditions, with the visual and auditory conditions showing an additional localized negativity. For the first component (C1), the V Only and AV conditions have an added focal negativity at lateral occipital electrodes, consistent with visual processing. In the second component (C2), the A Only and AV conditions have an added focal negativity over frontotemporal electrodes, consistent with auditory processing. Despite these two modality-specific aspects, the broader distributions of both C1 and C2 are mostly preserved across modalities, suggesting that C1 and C2 also capture supramodal responses. It is worth noting that ISC measured in each component is independently predictive of memory performance across subjects (C1: *r* = 0.38, *p* = 0.01; C2: *r* = 0.47, *p* = 0.001; C3: *r* = 0.35, *p* = 0.02, *N* = 43). Thus, our finding that synchronous activity across subjects predicts memory performance applies to supramodal and audiovisual evoked activity.

## Discussion

This study has two main findings. First, visual context enhances the memory of an auditory narrative despite lacking pertinent information, even when it is presented incongruently with the narrative (Fig. 2). This finding is notable since it applies to the biographical narratives that are commonly shared in everyday life experiences (New York Times and StoryCorp stories) rather than to stimuli constructed in the laboratory by experimenters. Our second finding is that subjects whose neural responses correlated more strongly with others had superior memories, consistent with results on the slower time scale of fMRI (Hasson et al., 2008; Fig. 4). While event-related potentials have been linked to retrieval (Paller and Wagner, 2002), no similar results are available for supramodal evoked responses, as we report here (Fig. 5). Importantly, our results extend previous findings using discrete stimuli (Murray et al., 2004; Thelen et al., 2012, 2014; Matusz et al., 2015) to the those of continuous and prolonged naturalistic stimuli and memory tasks. These results can be interpreted using theories of associative memory, the reliability of stimulus-induced encoding, and attentional engagement, as outlined below.

Since visual context enhanced memory for the auditory narrative in the absence of functionally informative content, our results lend support to the theory of associative memory wherein information retrieval is enhanced when it can be linked with a framework of associations (Yates, 1966; Paivio, 1991). In the unadulterated audiovisual case, the functionally uninformative visual was congruent and semantically linked to the audio story, with some clips containing specific visual clues associated with auditorily presented information. The coupling of semantically linked audio and visual information has been shown to augment overall comprehension of the material (Sumby and Pollack, 1954). However, previous research has found conflicting evidence regarding the role of a supplemental sensory modality in unisensory encoding (Thelen and Murray, 2013). Multisensory stimulation-dependent retrieval enhancements often depend on the meaningful congruency between the semantics of the auditory and visual content (von Kriegstein and Giraud, 2006; Matusz et al., 2015). A correspondence between sensory streams is thought to enhance the binding between them and therefore to induce a stronger memory trace (James, 1890). However, if coincident stimuli are incongruent or irrelevant, they may interfere with the ability to remember either stimulus individually (Cowan, 1999; Mayer et al., 2001; Matusz et al., 2015; Thelen et al., 2015) since human attention has a limited bandwidth (Broadbent, 1958; Sweller, 1994). Following this argument, it seems surprising that the temporally incongruent audiovisual condition (AVsc) was almost as effective as the unadulterated version (AV). It is possible, however, that information lingered in working memory, thus permitting an association between the two information streams despite their temporal misalignment (Cowan, 1999).

An alternative interpretation is that visual stimuli enhance the processing of auditory information and therefore augment memory. Neural activity was recorded to explore whether stimulus processing is predictive of memory performance. Similarly to memory performance, the addition of the visual modality increased the ISC above audio alone. While it could be a coincidence that visual stimulation independently affected memory and ISC, the increase in both memory and ISC when the congruent visual stimulus is added to the audio story is consistent with the interpretation that more reliable processing during encoding leads to better memory performance. Indeed, after controlling for the modality of the stimulus, narrative-evoked responses that were more correlated across subjects predicted improved recognition memory 3 weeks later (*r* = 0.49; Fig. 4).

The correlation of neural responses across subjects can only be high when responses are reliably reproduced in each individual. Thus, high ISC requires that each participant produces a reliable neural response to the stimulus. Based on the present results and previous literature, we argue that this reliability reflects the reliability with which each subject processes the material that they are presented with. The robustness of encoding has been linked to the reliability of evoked responses to repeated stimulus presentations in both animals and humans (Yao et al., 2007; Xue et al., 2010). Recent work in humans has also shown that repeat-reliability within individuals directly translates to the reliability of responses across subjects (Hasson et al., 2009; Byrge et al., 2015). High ISC may therefore represent faithful and repeatable auditory processing, which thus lead to enhancements in memory for auditory information. Indeed, for the A Only and AV conditions, C2 has a bilateral temporal distribution (Fig. 5) consistent with auditory cortex activity, and this component alone is a good predictor of memory performance. However, the most reliable component of the evoked response (C1) is also partially modality independent since its spatial distribution is similar, regardless of sensory modality (Fig. 5). This component may therefore also capture higher-level processing of the stimulus (Marinkovic et al., 2003). Due to its broad spatial topography, it may represent the engagement of diverse brain areas that are not solely sensorily driven. This component also independently predicts memory performance. This suggests that neural generators that are not specifically tied to auditory processing are an important part of the reliable stimulus processing that leads to memory formation.

It is possible that the level of attentional involvement with the stimulus corresponds to the extent to which the stimulus evokes synchronous responses across subjects, and that this synchronous activity therefore predicts memory performance (Posner, 1980; Luck et al., 1994; Fontanini and Katz, 2008). In this view, the ISC is modulated by the attentional engagement with the stimulus (Dmochowski et al., 2012). Consistent with this, recent work in our laboratory demonstrates that explicit manipulation of attentional state strongly modulates the level of ISC evoked by narrative stimuli (Ki et al., 2016), and attention is well known to affect learning and memory (Murdock, 1965; Baddeley et al., 1984). Similar to day-to-day experience, during incidental encoding, attention fluctuates since it is subject to a number of variables, including alertness, stimulus interest, and curiosity (James, 1890; Berlyne, 1966; Vuilleumier, 2005; Petersen and Posner, 2012). This inherent variability in attention over the course of the narrative may underlie the correlation between ISC and memory performance.

Visual context, regardless of its congruency, may therefore have aided in directing and maintaining attention to the auditory narrative, and this mediating variable, therefore, improved memory performance. To validate this interpretation, we analyzed the strength of oscillatory band powers, which have previously been associated with memory and attention (Klimesch et al., 1996; Foxe and Snyder, 2011). We anticipated that that alpha power would have an inverse relationship with memory performance (Klimesch et al., 1996; Hanslmayr et al., 2009). However, after controlling for the effect of modality, no significant relationship was found. It is worth noting that in the context of a naturalistic stimulus, alpha power may not be as sensitive to attentional modulation as ISC (Ki et al., 2016). It is also possible that the effect of alpha power modulation is too weak to correlate with memory performance after 3 weeks. Measuring other markers of memory, such as the modulation of evoked response magnitude (Paller et al., 1987), are unfeasible under the present circumstances, where individuals experience only one event, a single stimulus presentation. While ISC modulation suggests that attention played a role in memory performance, a conclusive link may require experiments where attentional state is explicitly controlled.

In conclusion, these experiments demonstrated memory enhancements when a functionally uninformative visual stimulus was added to an auditory narrative. This boost coincided with an increase in the correlation of narrative evoked responses across subjects. The extent to which individuals correlated with one another, thus processing the stimulus in a reliable and repeatable manner, predicted their memory performance. While ISC may be driven by modality-dependent stimulus features, this across-subject synchrony also exhibits a partially supramodal spatial pattern that may reflect encoding processes that induce subsequent memory. This measure of the reliability of neural processing may help to resolve the conditions under which adding extraneous information is beneficial to memory performance. It suggests that in a naturalistic setting where stimuli occur in a continuous stream, the reliability of processing may dominate other considerations, such as whether an additive stimulus is congruent or incongruent. Future studies should use additive supplemental stimuli that are either beneficial or detrimental to memory performance. We predict that the most relevant factor is how the added stimulus affects the reliability with which the relevant information is processed.

## Synthesis

Reviewing Editor: Daniela Schiller, Mount Sinai School of Medicine The decision was a result of the Reviewing Editor Daniela Schiller and the peer reviewers coming together and discussing their recommendations until a consensus was reached. A fact-based synthesis statement explaining their decision and outlining what is needed to prepare a revision is listed below. The following reviewer(s) agreed to reveal their identity: Avi Mendelsohn

The authors have addressed the concerns relatively adequately. There are still some points which I find problematic, perhaps this is common practice in EEG. While the authors could have addressed the concerns more thoroughly, as the main finding of the paper, that ISC correlates with memory across conditions, appears robust. A small note - saying that there is no effect of narrative with p=0.07 is misleading, even if it did not reach significance, that certainly indicates there may be a difference, however since the production did not interact with condition, this seems to hav no bearing on the results.
